# Therapy Resistance, Cancer Stem Cells and ECM in Cancer: The Matrix Reloaded

**DOI:** 10.3390/cancers12103067

**Published:** 2020-10-21

**Authors:** Kousik Kesh, Vineet K. Gupta, Brittany Durden, Vanessa Garrido, Beatriz Mateo-Victoriano, Shweta P. Lavania, Sulagna Banerjee

**Affiliations:** 1 Department of Surgery, Miller School of Medicine, University of Miami, Coral Gables, FL 33146, USA; kxk536@med.miami.edu (K.K.); vineetkgupta@med.miami.edu (V.K.G.); bxd324@miami.edu (B.D.); vtg11@med.miami.edu (V.G.); bxm740@miami.edu (B.M.-V.); shweta.lavania@med.miami.edu (S.P.L.); 2 Sylvester Comprehensive Cancer Center, University of Miami Hospital & Clinics, Miami, FL 33136, USA

**Keywords:** extra cellular matrix, cancer stem cells, cancer metabolism

## Abstract

**Simple Summary:**

The extracellular matrix (ECM) has emerged as a critical part of the tumor microenvironment. This glycoprotein- and proteoglycan-rich part of the tumor serves as a niche for the enrichment of cancer stem cells that can drive resistance to therapy and metastasis. Additionally, the ECM can act as a barrier to drug delivery, thereby physically contributing to resistance to therapy. This review summarizes the role of the ECM in enriching for cancer stem cells and how it contributes to therapy resistance in cancer. Finally, it discusses the attempts to develop molecules that can target the ECM as potential therapy options.

**Abstract:**

The extracellular matrix (ECM) has remained an enigmatic component of the tumor microenvironment. It drives metastasis via its interaction with the integrin signaling pathway, contributes to tumor progression and confers therapy resistance by providing a physical barrier around the tumor. The complexity of the ECM lies in its heterogeneous composition and complex glycosylation that can provide a support matrix as well as trigger oncogenic signaling pathways by interacting with the tumor cells. In this review, we attempt to dissect the role of the ECM in enriching for the treatment refractory cancer stem cell population and how it may be involved in regulating their metabolic needs. Additionally, we discuss how the ECM is instrumental in remodeling the tumor immune microenvironment and the potential ways to target this component in order to develop a viable therapy.

## 1. Introduction

The extracellular matrix (ECM) has long been considered as the inactive and physical component of any tissue. Specifically, its role in tissue regeneration and wound healing by providing the milieu for the adhesion, migration and differentiation of cells has been extensively studied and acknowledged over a period of time [1]. In pancreatic tumors, the ECM components like collagen and hyaluronan form a significant part of the tumor. They exert pressure on the blood vessels, constricting them and functionally impairing them, thereby creating a physical barrier which prevents effective drug delivery, thereby contributing to therapy resistance [2,3,4]. However, it is not clear whether the ECM components affect intra-tumoral and microenvironmental factors that lead to chemo- and immune resistance. One of the key contributors to therapy resistance is the treatment-refractory population of cancer stem cells (CSCs) within the tumors that are selected and enriched upon interaction with the microenvironment [5,6]. CSCs are defined as undifferentiated, quiescent cells that exist as a minority subpopulation within the entire tumor mass and have a potential to form the bulk of the tumor tissue even from a single cell. Whether the ECM components of the microenvironment play any role in influencing the enrichment of these CSCs in the tumor remains an enigma. In this review, we focus on how the ECM provides a favorable niche for the enrichment of CSCs and modulates the immune microenvironment and whether it can be targeted to sensitize pancreatic tumors to chemo- and immune therapy.

## 2. Extracellular Matrix as a Niche for Cancer Stem Cell Formation and Maintenance

The cancer stem cells (CSCs) are a population within tumors that have been classically defined as cells with the capability of self-renewal, resistance to conventional therapy and the capability of metastasis. The tumor microenvironment and particularly the ECM has emerged as a favorable niche for cancer stem cell (CSC) enrichment. In the following section, we will review how the cellular and the acellular components of the niche select for the CSC population within a tumor and govern its function.

### 2.1. Enrichment of CSC Population within the Tumor

Among the various microenvironmental factors are the stromal fibroblasts that secrete the pro-inflammatory cytokines, which in turn activate oncogenic transcription factors within the tumor epithelial cells. These transcription factors (STAT3, NF-kB) often trigger a cascade of events that trigger self-renewal pathways regulated by Sox2, Oct4 and Nanog1, thereby promoting a stemness-associated phenotype [7,8]. Our research in pancreatic cancer (currently under revision) shows that stromal cells secrete IL6 which activates the STAT3 signaling pathway within the tumor cells which invariably leads to the enrichment for stemness. Acellular components of the microenvironment, such as hypoxia, trigger transcriptional activity through the Hypoxia Inducible Factor 1 (HIF1) signaling pathway and promote activation of stemness-associated transcription factors [9]. Recent and emerging research from various groups reveal that the CSC population within a tumor is a dynamic and plastic population that is constantly changing between the non-CSC and the CSC state based on the fluctuating cues within the tumor microenvironment [10,11]. Upon conditions of nutritional and oxidative stress (as is common in a hypoxic tumor), certain cells within the tumor activate the enhanced survival pathways to help tide them over in the unfavorable conditions. This is manifested in the CSCs by enhanced resistance to therapy along with high metastatic potential. A recently published study from our group shows that under nutritional deprivation or hypoxia or due to chemotherapy treatment, the CD133 + CSC population markedly upregulates the long-noncoding RNA GAS5 [12]. This promotes quiescence in this population of CSCs, thereby helping these cells to survive during these stressful conditions. Additionally, CD133 + CSCs also have their metabolites routed through the biosynthetic pathways. Thus, the upregulation of GAS5 and suppression of proliferation, along with key metabolites in the biosynthetic pathways, puts these cells in a uniquely advantageous position in which they can regain their aggressive proliferative potential as soon as these adverse conditions recede [12].

### 2.2. ECM and Stem Cell Homeostasis

As an integral part of the microenvironmental niche, the ECM components play an important role in maintaining stem cell homeostasis [13]. In fact, decellularized natural ECM scaffolds have been shown to guide stem cell differentiation into cell types from which the ECM is derived [14]. This property of the ECM is widely used for tissue engineering in the case of organ damage. Additionally, it is well established that the CSCs can reside in niches within the microenvironment [15] Moreover, the ECM niche provides structural support to the tissue and widely influences cell behavior [16]. The impact on the functional properties of CSCs is mediated by the interaction of the ECM with the cell surface receptors, specifically the integrin pathways [17,18,19].

### 2.3. ECM–Integrin Interaction and Stemness

The ECM also plays a crucial physical role in anchoring the CSCs to the niche. For instance, in acute myeloid lymphoma (AML) cells, the interactions of the stem cells with the integrin have been reported to regulate drug sensitivity [20]. The ECM plays a crucial role in the maintenance of the CSC plasticity [21] by regulating integrin-mediated signaling. Integrins present on the surface of tumor and stromal cells are the primary receptors involved in cell–matrix adhesion and play a profound role in the ability of the CSCs to survive in specific locations. However, in some cases, these receptors can also function in the absence of ligand binding to promote stemness and survival in the presence of environmental and therapeutic stresses [19]. 

Many integrins that are enriched in the normal adult stem and progenitor cells are also markers of CSCs, including integrin subunits β1, α6 and β3 [22]. Among these, α6 is the most widely observed, enriching for CSCs in breast, prostate, squamous cell carcinoma and colorectal cancers [23,24,25,26]. Integrin β4 has been identified as a CSC marker in lung cancer, where it mediates self-renewal, tumor propagation and chemoresistance [27]. Apart from serving as CSC markers, integrins also enhance the cancer stem cell functions. Notably, in glioblastoma, integrin α6 is involved in the maintenance of cancer stem cells, while in breast cancer, it promotes tumor initiation by regulating the induction of the Polycomb complex protein BMI-1 [28,29]. Thus, the ECM–integrin interaction in the microenvironment can profoundly regulate CSC functions in a tumor by influencing both the autocrine and the paracrine signaling pathways.

### 2.4. Role of Collagen in CSC Enrichment and Function

The tumor ECM is quite unique in nature as compared to the normal cell ECM as it overexpresses many unusual collagen fibrils with abnormal crosslinking that affects the cancer stemness by activating several signaling cascades. Type 1 collagen plays an important role as a scaffold of the CD133-positive GSCs and accelerates cell invasion through the PI3K/Akt/Giardin pathway [30]. A study by Kirkland et al. showed that type 1 collagen impedes differentiation and promotes expression of CD133 and Bmi1 (stem cell markers) in colorectal cancer. It also increased the Pancreatic adenocarcinoma (PDAC) tumor initiating potential, self-renewal, and enrichment of CSCs through the activation of Focal Adhesion Kinase (FAK). COL1A1 confers a survival advantage and enhanced Hepatocellular Carcinoma (HCC) cell clonogenicity, tumorsphere formation and expression of stemness genes like SOX2, OCT4 and CD133 [31]. In addition, another study published by Li et al. showed that collagen facilitated the stemness and metastasis in colorectal cancer through an integrin/PI3K/AKT/Snail signaling pathway [32]. Mammary tumors overexpressing COL1A1 displayed higher CSC activity, enhanced AKT-mTOR and Yes-associated protein (YAP) activation along with an increase in metastases [33]. 

**Figure 1:**
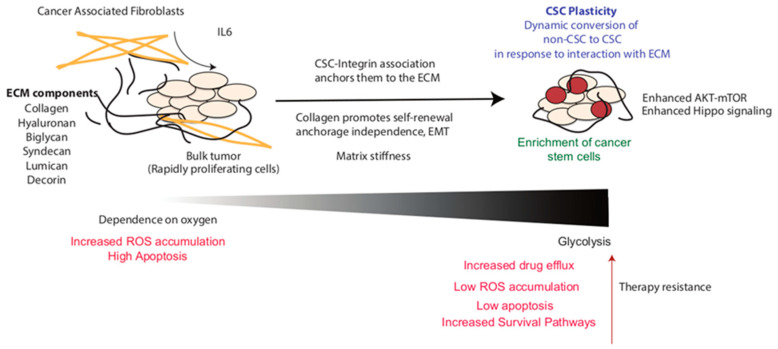
Enrichment of cancer stem cells in the presence of the extracellular matrix (ECM) in the tumor microenvironment. Interaction with the ECM upregulates self-renewal and signaling pathways and induces metabolic reprogramming to enrich therapy-resistant cancer stem cell populations.

Abnormal collagen crosslinking generates mechanical pressure that increases the rigidity and stiffness of the cancer cell matrix. The matrix stiffness is an important factor that is transmitted to the CSCs and regulates their proliferation and plasticity. For instance, in colorectal cancer, the matrix stiffness mediates cancer cell stemness via the YAP pathway. Specifically, the expression levels of the stemness markers CD133 and Lamin A/C are markedly increased due to YAP activation which occurs in response to the increase in the matrix rigidity [34]. Similarly, chemoresistance and stemness of Hep-2 cells increases via Sox2-mediated ABCG2 overexpression with an increase in the matrix stiffness [35], while in breast cancer stem cells, matrix stiffness promotes development by modulating integrin-linked kinase (ILK), as summarized in Figure 1 [29]. 

### 2.5. Proteoglycans and CSC

Proteoglycans (PGs) are glycoproteins of the ECM composed of a core protein and one or several covalently attached sulfated glycosaminoglycan chains [36]. Cell surface PGs integrate with various cytokine and chemokines in the tumor microenvironment and activate multiple signal cascades including Notch, Wnt and hedgehog in CSCs [37]. The two major heparan sulfate proteoglycan families are those of Glypican and Syndecan. Several studies have revealed a functional role of transmembrane syndecan-1 (SDC-1) in CSCs. SDC-1 is a molecular marker for triple negative breast cancer and regulates the cancer stem cell phenotype by affecting IL6/STAT3, Notch and EGFR pathways [38]. Loss of Sdc-1 in the human breast cancer cell lines resulted in a significant reduction of several stemness-related phenotypic characteristics, including the side population (SP) phenotype, ALDH-1 activity, the CD44(+)CD24(−/low) phenotype and the capacity to form three-dimensional spheres under nonadherent cell culture conditions. Glypican is overexpressed in liver cancer cells and regulates CSC self-renewing ability by regulation of autophagy [39]. GPC4 overexpression increased 5-fluorouracil (5-FU) resistance and pancreatic cancer stemness through the activation of the Wnt/β-catenin pathway [40]. Small leucine-rich proteoglycans (SLRPs) like Lumican and dDecorin were overexpressed in glioblastoma and neuroblastoma CSCs and provided chemoresistance and stemness properties [41], while biglycan, another SLRP, confers chemoresistance to HCC by NF-kB activation [42].

### 2.6. Hyaluronan and CSCs

Hyaluronan (HA), a glycosaminoglycan, is ubiquitously found in the ECM in cancer cells and contributes majorly to their proliferation, plasticity, differentiation and enrichment. CD44 is a transmembrane glycoprotein known to act as a receptor for a wide variety of ECM ligands, such as HA and activates an array of signaling pathways upon interaction. HA serves as a primary ECM component of the stem cell niche and is often overexpressed in several cancer types. In fact, the HA-rich ECM provides a favorable microenvironment for self-renewal and maintenance of CSCs by influencing the behavior of stromal cells [43]. In a study by Okuda et al. [44], HA in metastatic breast cancers stimulated the interactions of CSC and tumor-associated macrophages (TAMs) that, in turn, activated stromal fibroblasts to support self-renewal via fibroblast growth factor (FGF) activation. Hyaluronan synthase (HAS2) has also been shown to be instrumental in breast CSC-mediated metastasis [44]. Excessive HA accumulation increases cancer stem cell enrichment through the coordinated regulation of “Twist” and transforming growth factor β (TGF-β)-Snail signaling [45]. Furthermore, HA-CD44 binding promotes the Nanog–Stat3 interaction in head and neck, breast and ovarian cancer and induces stemness and chemoresistance [46]. 

## 3. Extracellular Matrix in Cancer Stem Cell Migration and Metastatic Niche Regulation

The ability of cancer cells to evade therapy, extravasate from the primary tumor site and to colonize a secondary metastatic site are some of the factors that majorly contribute to cancer metastasis. These cancer stem-like cells are a minority cell population within a tumor that are majorly responsible for cancer cell metastasis. Like the primary site, CSC metastasis is also driven by the niche formation at the metastatic site [47,48,49]. 

Cell–cell interactions between CSCs and cells of the microenvironment, along with cancer-associated fibroblasts (CAFs) and immune cells, as well as the cell–matrix interactions, all contribute to the CSC migration and establishment of metastatic sites. In recent years, there has been a renewed focus on understanding why certain primary cancers have the ability to metastasize to preferred secondary sites. For example, breast cancer cells predominantly metastasize to the lungs and pancreatic cancer cells predominantly metastasize to the liver. In other words, is there an inherent host mechanism that attracts these specific cancer types to these metastatic sites, or do the primary cancer cells have mechanisms that prime these secondary locations to create CSC niches that allow for the proliferation of the CSCs, which eventually leads to metastasis? Any insights into these important queries will result in a renewed understanding of the factors which influence proliferation and metastasis of CSCs in many types of cancers [47,50,51].

In recent years, specific ECM molecules, including tenascin-C, have become an intense focus of investigation. Increasing interest in identifying the mechanism of metastatic dissemination to specific secondary sites identified tenascin-C as a key molecule in directing breast cancer cells specifically to lung tissue as a site of metastasis [52,53]. Another major contributor to the metastatic niche formation is the existence of extracellular vesicles or, more specifically, exosomes containing ECM-modulating genes. Additionally, yet another area of interest in understanding the interaction of the ECM and CSCs in metastatic niche formation is identifying the importance of the ECM in the epithelial to mesenchymal transition (EMT). One hallmark of malignancy is the reprogramming of epithelial cells into a more mesenchymal phenotype. In lung cancer, ZEB1 has been described as a transcription factor that plays a major role in this EMT [54]. Furthermore, in ZEB1-activated mesenchymal lung cancer, increased collagen deposition is a direct consequence of increased collagen-associated genes [55]. This collagen in metastatic sites was found to be more organized and linear than in primary lung tumors due to the expression of the Lysyl oxide (LOX) family of collagen crosslinking genes. These crosslinked collagens also activate FAK/Src signaling in the mesenchymal cancer cells which promotes migration and invasion of these cells. In cervical cancer, a recent study showed that integrin α3 is overexpressed in the cervical cancer cell line SiHa [56]. Knockdown of integrin α3 in this cell line had no effect on the proliferation but did inhibit cell motility and migration. Conversely, when overexpressed in C33A cells, it led to an increased migration of these cells, suggesting an important role of integrin α3 in metastasis of cervical cancer [56].

In pancreatic cancer, the role of ECM, specifically hyaluronan (HA), has been extensively studied (as discussed in the earlier section). Among these, CD44 (one of the CSC markers in pancreatic cancer) is a hyaluronan receptor and has been shown to activate signaling pathways leading to metastasis [57]. The role of environmental metabolites in reshaping the microenvironment of the metastatic niche has also been recently acknowledged and is an intense area of research. For instance, in breast cancer, pyruvate drives the collagen-based remodeling in the lung metastatic niche by upregulating alpha ketoglutarate synthesis which, in turn, affects the collagen hydroxylation [58].

## 4. Regulation of Cancer Stem Cell Metabolism by ECM

### 4.1. Hypoxia in Regulation of ECM and CSC Metabolism 

The ECM not only provides physical support to the cells but also dynamically influences tumor metabolism [59]. In breast and pancreatic cancer, localization of the desmoplastic fibrotic areas often coincides with the hypoxic region of the tumor [6,60]. Meanwhile, the cancer cells adapt to this low oxygen availability by increasing the transcriptional activity of hypoxia-inducible factors, namely HIF 1 and 2, which, subsequently, reprogram the cancer cells by regulating the expression of multiple genes involved in the angiogenesis to increase the blood flow and by rewiring the glucose metabolism to efficiently utilize the scarcely available nutrients [61]. Hypoxia regulates cancer stem cell “niches” through direct activation of HIF and its target genes. Recently, HIFs have also been shown to regulate specific signaling pathways and transcription factors, including Oct4 and Notch, which are critical in maintaining stem cell self-renewal and multipotency. Recent studies in pancreatic cancer published by multiple groups have shown that high expression of HIF1α under hypoxia promoted CSC-like features in pancreatic cancer cells by inducing the CSC marker expression, chemoresistance and EMT phenotype [5,62,63]. Similarly, hypoxia-mediated HIF1α also induces stem cell-like characteristics in ovarian cancer cells [9]. Additionally, hypoxia and HIF1 have been shown to regulate ECM remodeling and promote fibrosis in liver, kidney and adipose tissue [64,65,66]. Kidney, skin and heart fibroblasts cultured under hypoxic conditions showed an increase in type I procollagen α1 mRNA levels [67,68,69]. Interestingly, HIF1 not only regulates the ECM gene expression but also regulates their post translational modification by modulating the expression of genes encoding collagen prolyl and lysyl hydroxylases. Specifically, P4HA1 and P4HA2 are prolyl hydroxylases which are required for collagen deposition, whereas PLOD1 and PLOD2 (lysyl hydroxylases) catalyze lysine hydroxylation of collagen which provides stiffness to the ECM [70]. HIF1 regulates the expression of PLOD1, PLOD2, P4HA1 and P4HA2 enzymes in cancer cells, fibroblasts and endothelial cells [62,67,71]. In breast cancer and stromal cells, small hairpin RNA (shRNA)-mediated knockdown of HIF1α expression inhibits collagen deposition in vitro [70,71]. Apart from collagen deposition, the collagen degradation also plays a crucial role in the ECM remodeling in hypoxic environments by upregulating the expression of several families of proteinases, like matrix metalloproteinases (MMPs), which degrade the ECM and mediate cancer cell invasion and tumor metastasis [72,73,74].

Metabolic reprogramming and adaptation are believed to be crucial hallmarks of cancer stem cells. Under normal conditions, differentiated non-cancer cells predominantly rely on oxidative phosphorylation (OXPHOS) as their main source of energy. However, cancer cells rely on glycolysis as their preferred energy source using a phenomenon called the Warburg effect, named after the German biochemist, Otto Warburg, who discovered that cancer cells used a different metabolic pathway than normal cells [75]. Multiple peer-reviewed publications from our group corroborate these observations as well [6,7,12]. Interestingly, the metabolic phenotype of CSCs appears to vary across different tumor types and tumor microenvironments. For instance, multiple studies in osteosarcoma, breast cancer, colon cancer and ovarian cancer suggest that the CSCs use aerobic glycolysis as their preferred energy source [76,77,78]. Emerging studies also suggest that the preferred energy source of CSCs in glioblastoma, lung cancer and leukemia is mitochondrial oxidative metabolism [79,80]. However, CD133 + CSCs isolated from pancreatic tumors of spontaneous KRAS^G12D^ TP53^R172H^PDX (KPC) mice revealed a glycolytic dependence for their energy requirement. Glucose serves as an essential nutrient for CSCs for their energy requirements and its presence in the tumor microenvironment significantly upregulates CSC numbers in the tumor. Additionally, the presence of glucose upregulates the expression of genes associated with the glucose metabolism pathway, such as HK-1, HK-2, c-Myc, Glut-1, PDK-1 etc., which further contributes to the increase in the CSC population in the tumor [81]. Furthermore, glucose deprivation or glycolysis inhibition results in a proportional decrease in the CSC population [82]. Contrastingly, in prostate cancer, the stromal cells rely on a “reverse Warburg effect”, where the cancer-associated fibroblasts use aerobic glycolysis as a source of energy and secrete lactate which is taken up by the cancer cells to generate energy using oxidative phosphorylation (OXPHOS) [83].

### 4.2. ECM Regulated-Metastasis in CSCs

**Figure 2:**
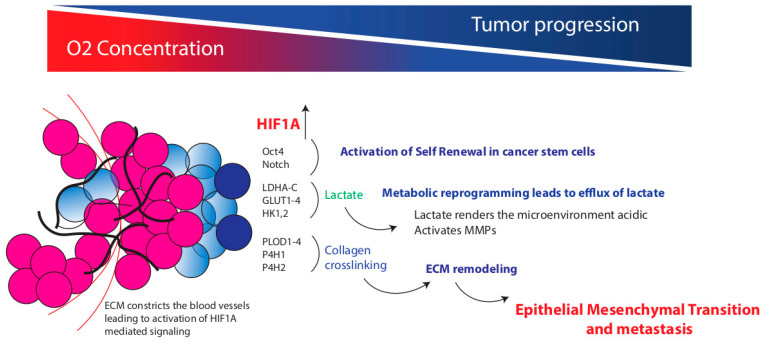
Regulation of cancer stem cell (CSC) metabolism by ECM. Deposition of ECM during tumor progression leads to extensive hypoxia within the tumors. This activates HIF1A-mediated signaling in the tumor cells, resulting in activation of self-renewal pathways, metabolic reprogramming and ECM remodeling, eventually leading to metastasis.

CSCs are known to play a crucial role in cancer metastasis since they have a greater capacity to migrate and invade [63]. Cell migration is an energetically costly event involving the remodeling of cell to cell and cell to ECM interactions, ECM degradation, focal adhesions, the formation of invadopodium and epithelial to mesenchymal transition (EMT) transition [84]. The glycolytic dependence of CSCs plays a crucial role in this cancer metastasis, since several glycolytic enzymes drive the formation of invadopodium structures, cellular protrusions and ECM degradation, resulting in cancer cell migration and invasion [85]. Moreover, the release of lactate, a final product of glycolysis, alters the extracellular pH and facilitates the ECM degradation, resulting in cancer cell migration [86]. Similarly, methylglyoxal, a glycolytic byproduct, activates the Yes-associated protein (YAP) signaling pathway and induces EMT in breast cancer cells [87]. Interestingly, mitochondrial metabolism can also modulate ECM remodeling and promote cancer cell invasion in melanoma and ovarian cancer [88,89]. Recent work has shown that the depletion of hyaluronan by hyaluronidase induces GLUT-1 expression in cancer cells which, in turn, triggers glucose uptake and an increase in the rate of glycolysis, all of which eventually lead to a concomitant acceleration of cell migration [90] (Figure 2).

## 5. Cancer Stem Cell-Derived ECM Maintains Immune Evasion

**Figure 3:**
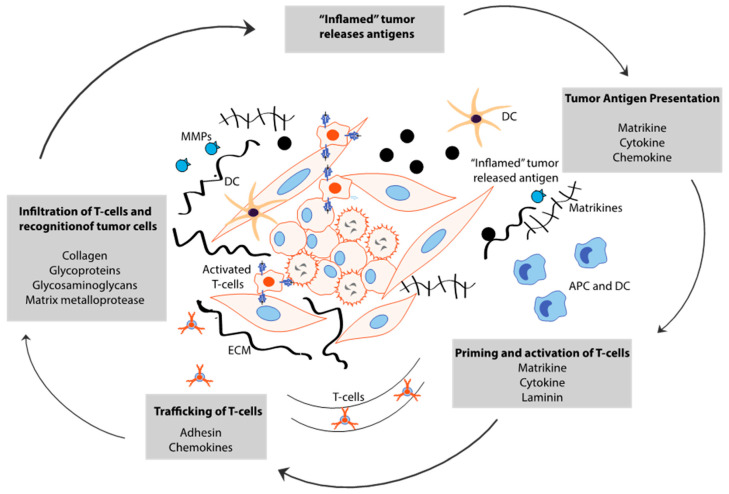
Role of the ECM in modulating the tumor immune microenvironment: Matrix remodeling shapes the inflamed immune microenvironment. “Inflamed” tumor releases the antigens that are used for antigen presentation. The ECM components are processed by the metalloproteases to release matrikines (versikine) which in turn regulate the priming and activation of T cells. ECM also serves as a scaffold for T cell migration by haptotaxis.

The immune cells within the tumor microenvironment (TME) play a key role in cancer progression and treatment. The balance between the effector and tolerogenic immune response dictates tumor fate and the myriad cellular interactions in the TME determine whether the immune cells may possess anti-tumor or tumor-promoting functions [91,92]. It is known that the individual components of the ECM and its three-dimensional structure and biophysical properties can also modulate the essential immune functions, such as migration, immune cell activation and proliferation [93]. However, while the role of the ECM in tumor progression has been extensively studied, the crosstalk between immune cells and ECM tends to be neglected. Cytotoxic T lymphocytes (CTLs) play an important role in eliminating cancer cells in an antigen- and cell contact-dependent manner. However, they are often found trapped in the dense ECM compartment within the TME. Lymphocytes that are first attracted to the tumor site by cytokine gradients (chemotaxis) are often diverted from this direction upon contact with areas of increased stiffness, thus restricting the migration of these cells to the tumor core [94,95]. Besides acting as a physical barrier, the constant ECM remodeling and overexpression of certain matrix components also facilitate the recruitment of myeloid cells, which, eventually, get polarized toward phenotypes that support tumor proliferation and invasion, ECM remodeling and CTL suppression [96,97]. Increased collagen density has been shown to enhance macrophage and neutrophil recruitment and promote tumor growth in breast cancer [98]. Additionally, during tissue inflammation, ECM proteins are degraded by proteases into bioactive matrix fragments, sometimes referred to as matrikines, which can have chemoattractant properties and pro-inflammatory effects similar to some cytokines [99,100,101,102]. Moreover, the ECM components hyaluronan and versican can bind to Toll-like receptors 2 and 4 to induce inflammatory gene expression in a variety of immune cells contributing to fueling inflammation at tumor sites [103,104,105]. Tumor-associated immunosuppressive cells secrete cytokines and other inflammatory mediators that support stemness properties, metastatic potential and tumorigenicity in CSCs [106]. Therefore, CSCs design their own microenvironment by overexpressing several matrix components and secreting signals that alter the function of tumor-specific CTLs, and promote the expansion of immunosuppressive, pro-tumorigenic cells [107]. For instance, overexpression of tenascin-C (TNC) in the stem cell niche has been demonstrated to protects prostate stem-like cells from immune surveillance via the suppression of T cell receptor (TCR)-dependent T cell activation. The interaction of TNC with α5β1 integrin on the T cell surface blocks the actin-based cytoskeleton reorganization which is required for optimal T cell activation after TCR stimulation [108]. Additionally, exosomes containing TNC were shown to be secreted by brain tumor CSCs, inhibiting T cell proliferation through interaction with integrins on T cells and reduced mTOR signaling. Accordingly, circulating exosomes from glioblastoma patients were found to have increased TNC expression and T cell suppressive activity than those from control individuals [109]. Several studies indicate a particular influence of CSCs in driving the recruitment and polarization of macrophages within the niche. Additionally, tumor-associated macrophages (TAMs) protect and promote CSC functions in the tumor microenvironment. Altogether, the TAMs provide crucial signals to promote CSC survival, self-renewal, maintenance and migratory ability, and in return, CSCs deliver the tumor-promoting signals to TAMs that further promote tumorigenesis (Figure 3). 

Taken together, these observations support a significant role for ECM remodeling in promoting immune cell recruitment and inflammation in the tumor microenvironment and represents an innovative therapeutic target.

## 6. Targeting Extracellular Matrix to Remodel CSC Niche

One of the hallmarks of cancer stems cells (CSCs) is their ability to induce tumor relapse and resistance to standard chemotherapy treatment strategies. CSCs depend on their niche for essential support to grow and survive. Thus, disrupting CSC niches could potentially inhibit their growth, ultimately improving patient survival [110]. The ECM was traditionally considered to merely act as a scaffold, but in recent times, its role in CSC survival and maintenance has become quite evident. The physical properties of the ECM, like the rigidity, porosity, insolubility and direct or indirect signaling pathways of the ECM can influence resident cells’ biological function [16]. The ECM receptors for CSCs not only provide anchorage but also help by mediating paracrine signaling involved in self-renewal and differentiation. The transformed cells with stem cell-like properties compete with the normal cells within the niche and eventually manifest as dormant clones [111]. Therefore, future therapies targeting the CSC environment/niche as well as CSCs directly may be suitable pathways for further investigation [110].

It has been previously shown in breast, ovarian and head and neck cancers that acquisition of CSC characteristics can occur by the hyaluronan acid (HA)–CD44 interaction. As a result, this increases the expression of stemness (e.g., NANOG and SOX2) and drug resistance factors (MDR1). HA synthesis is accomplished by hyaluronan synthetase 1–3 (HAS1-3) and their expression levels correlate with poor prognosis. 4-Methylumbelliferone (4-MU) is an approved drug for bile therapy and inhibits HA synthesis [95]. In an in vitro study using mammary carcinoma cells, Henke et al. observed a decrease in intracellular HA accumulation as well as in the ECM [95]. Additionally, 4-MU-induced loss of the HA receptor CD44 reduced cell migration and invasion [112]. Furthermore, in vivo studies using prostate and pancreatic cancer murine tumor models, treatment with 4-MU also reduced HA accumulation [112]. Reduced CD44 activation led to decreased PI3K signaling and AKT and ERK phosphorylation [113]. In vivo studies using a murine pancreatic ductal adenocarcinoma (PDAC) model showed that the treatment with PEGylated human recombinant PH20 hyaluronidase (PEGPH20) reduces HA content and improves gemcitabine and DOX [114]. Additionally, a phase 3 clinical trial, in which patients with hyaluronan stage 4 PDAC were given PEGPH20 with nab-paclitaxel plus gemcitabine failed to improve patient outcome [115]. Thus, understanding this bidirectional crosstalk between CSCs and their niche is critical in understanding and overcoming the therapeutic resistance. Previous studies in pancreatic cancer have shown that reducing the stroma by targeting HA leads to increased efficacy and delivery of standard chemotherapy, suggesting that both CSCs and cancer cells can be targeted [19]. Thus, it is safe to predict that future studies must target both cancer cells and the CSC niche in order to demonstrate successful outcomes. 

The CSC–TME interaction controls the plasticity and functionality of CSCs. This contributes to their heterogeneity within the tumor. The presence of this mixed population of CSCs further impedes the efficacy of targeting drugs and efforts must be made to understand the factors contributing to their increase within the tumor [116]. The ECM can be remodeled significantly by CSCs to promote their survival via release of growth factors or cytokines. Specifically, collagen has been shown to activate multiple transcriptional programs to induce CSC self-renewal and preserve stemness and increased collagen production is observed in the stromal part of the tumor [32]. The family of enzymes of Lysyl hydroxylases (LOX) is also highly expressed in the stroma and is necessary for the processing and secretion of collagen [117]. The highly crosslinked collagenous matrix leads to a rigid tumor stroma and impedes effective drug delivery [95]. In murine breast cancer models, inhibition of Lysyl oxidases using 2-aminopropionitrile reduced collagen deposition and sensitized the tumors to doxorubicin (DOX), thus reducing tissue stiffness [118]. However, in a pancreatic cancer phase 2 trial, the addition of Simtuzumab, a LOXL2 antibody, in addition with gemcitabine, did not provide encouraging results [119]. This could be due to the fact that targeting LOXL2 specifically does not inhibit the biological activity of other LOXL variants that are invariably present in the microenvironment milieu. Therefore, future studies should explore molecules that target all members of the of the LOXL family broadly [120].

Presently, multiple clinical trials involving novel or previously known inhibitors are being conducted in different clinical settings targeting different aspects of ECM-induced cancer stemness. For example, STAT3 inhibitor BBI-608, Napabucasin, in addition to *nab*-paclitaxel and gemcitabine, is being tested in a phase III clinical trial in patients with metastatic pancreatic adenocarcinoma (NCT02993731). WNT and β-catenin signaling play an important role in antitumor activity and the small molecule β-catenin inhibitor CWP232291 is being investigated in prostate cancer cell lines as well as cells derived from Castration Resistant Prostate Cancer (CRPC) patients. Recently published studies by Pak et al. have shown that CWP232291 has antitumor activity via upregulation of C/EBP homologus protein (CHOP) and suppressing β-catenin expression via WNT pathway modulation [121]. Thus, it is quite imperative to further elucidate the role of ECM components as ECM molecules may play different roles in a context-dependent manner, be it acting as a physical barrier to drug delivery or by promoting differentiation of normal cells to cancer stem cells [19].

The tumor microenvironment promotes chemoresistance by maintaining the phenotype of CSCs. Collagen, via integrin signaling pathways, promotes CSC self-renewal in addition to serving as a physical barrier. JNK signaling may play a crucial role in establishing the CSC niche and by regulating the crosstalk within the niche that further contributes to chemoresistance and metastasis. Insua-Rodriguez et al. demonstrated that JNK signaling promotes upregulation of ECM protein genes. They also demonstrated, in a breast cancer mouse model, that treatment with JNK inhibitor CC_401 disrupted expression of ECM proteins and enhanced the effect of paclitaxel, suggesting that JNK signaling is involved in modulating the niche that allows CSCs to evade chemotherapy and promote metastasis. Thus, the JNK pathway provides a target to modify the CSC microenvironment and improve patient response to cancer therapy [122].

Another avenue that has been pursued is targeting the matrix metalloproteinase family of proteases (MMPs) to alter the ECM stiffness. Normally, MMP9 is activated in response to an external insult. In cancer, MMP9 facilitates the invasiveness and metastatic phenotypes of tumor-supporting cells. In addition, the overexpression of MMP9 increases the invasiveness of certain cancer cell lines and is involved in disease progression [15]. However, one important thing to consider while targeting the MMPs is that due to their context-specific effects, it is important to develop therapeutics for the right pathological state. Clearly, this redundancy is the reason why the MMPs that were pursued clinically failed (phase 3 clinical trial) to overcome cancer metastasis in patients. These failures indicate an urgent need for further characterization of the MMP inhibitors and their complexity.

Targeting integrin-mediated signaling pathways is yet another strategy to target cell–ECM communication. Disrupting ECM mechano-sensing and, consequently, disrupting the signals incoming from the extracellular or intracellular environment by using integrin inhibitor Cilengitide is one such viable approach [110]. The complex interactions between the ECM–tumor–stromal fibroblasts appear to be the major reason why just ECM targeting has not been very successful as a therapy option. However, these interactions are being acknowledged and appreciated by biologists. The detailed understanding of the crosstalk between ECM proteins, glycans with cancer-associated fibroblasts and cancer cells will eventually help in designing better combinations that can be effective in cancer treatment.

Among the other metabolic inhibitors, a recent study from our group has shown that targeting glutamine metabolism and the hexosamine biosynthesis pathway profoundly affects the ECM as well as self-renewal in pancreatic tumors [123]. Lastly, carbonic anhydrases (CAIX) have been shown to expand the CSC population during hypoxia. Thus, inhibiting CAIX may be yet another strategy that needs to be further explored in this context.

To summarize, it is obvious that further studies are needed to understand the plasticity of CSCs and their ability to adjust their response in the face of environmental stressors. Hypoxia is an important feature of the CSC niche, especially in solid malignancies, wherein, apart from the glucose-based metabolism, the hypoxic environment may favor the transformation of cancer cells towards a stem-like phenotype which allows them to utilize several sources of energy. These stem cells are thus capable of adapting to the rapidly changing environmental milieu and eventually thriving by maximizing their survival potential using multiple metabolic strategies. Thus, any efforts towards targeting these CSCs must incorporate evaluating multiple angles at once [124]. 

## 7. Conclusions

CSCs and normal stem cells share many targetable factors, resulting in challenges regarding the therapeutic window. CSC–microenvironment interactions are important for maintenance and progression. Due to the dependence of CSCs on their niche, it is plausible that microenvironment-targeted strategies will be effective in developing anti-CSC therapies. The ECM has been acknowledged as a niche for CSC populations in cancer. ECM targeting has shown promise in overcoming therapy resistance by improving drug delivery as well. Thus, the role of the ECM in remodeling the microenvironment in a dynamic manner needs to be studied in greater detail to develop viable therapeutic strategy in resilient cancers like pancreatic cancer.
